# Acute Pancreatitis During COVID-19 Pandemic: An Overview of Patient Demographics, Disease Severity, Management and Outcomes in an Acute District Hospital in Northern Ireland

**DOI:** 10.7759/cureus.18520

**Published:** 2021-10-06

**Authors:** Bakhat Yawar, Ahmed Marzouk, Heba Ali, Ayeisha Asim, Tamer Ghorab, Zahid Bahli, Mohammad Abousamra, Samara Fleville

**Affiliations:** 1 General Surgery, The Western Trust Health & Social Care Jobs in Northern Ireland (HSCNI) (Altnagelvin Area Hospital), Derry/Londonderry, GBR; 2 Radiology, The Western Trust Health & Social Care Jobs in Northern Ireland (HSCNI) (Altnagelvin Area Hospital), Derry/Londonderry, GBR; 3 Geriatrics, The Western Trust Health & Social Care Jobs in Northern Ireland (HSCNI) (Altnagelvin Area Hospital), Derry/Londonderry, GBR

**Keywords:** acute pancreatitis complications, digestive organs/ liver/ pancreas, pancreatitis outcomes, covid-19 impact on general surgery, pancreatitis demographics

## Abstract

Background

Acute pancreatitis (AP) is a common disease requiring admissions under surgical and critical care units. The two most common causes are alcohol and gallstones. Coronavirus disease 2019 (COVID-19) pandemic had a significant impact on service delivery and patient management throughout all surgical specialties. In this study, the primary aim was to ascertain the incidence of COVID-19 in acute pancreatitis patients. Secondary objectives were to study aetiology, demographics, severity, 30-day mortality, outcomes and management of acute pancreatitis patients from 1st March, 2020 till 31st August, 2020.

Methods

A retrospective observational review of all patients admitted under the General Surgical team was performed. Information regarding demographics, severity of AP (using Glasgow score, Atlanta classification and CT severity index score), ICU admission and organ support, treatment modalities and follow-up data for outcomes was collected based on data collection tool used by COVID-PAN study and results were compared to outcomes results of COVID-PAN study.

Results

Forty-three (43) patients were admitted with AP. Only one patient (2.3%) was diagnosed with COVID-19 at the time of pancreatitis. Gallstones were noted to be the most common cause of AP in our population. Mortality was 7% (3 patients). Five patients (11%) needed ITU admission due to organ dysfunction. Three patients (7%) developed ARDS.

Conclusion

The overall incidence of COVID-19 in pancreatitis in our population of the study was low. The incidence of COVID-19 during the first wave in Derry/Londonderry area was low and this may explain why the incidence was low in our study as well. Patients with AP in our target population were mostly elderly, one in five had moderate to severe or severe pancreatitis and in 16.3% the aetiology could not be identified. As observed in other centres globally, urgent cholecystectomy for gallstone pancreatitis faced significant delays with no patients being offered index cholecystectomy and only 4 out of 19 patients having undergone interval cholecystectomy within six months of index admission for gallstone pancreatitis in our centre.

## Introduction

Acute pancreatitis (AP) is characterised by inflammation of pancreas, damage to acinar cells and may be associated with local or systemic complications. It occurs due to obstruction of pancreatic ducts, activation of pancreatic enzymes such as trypsin and autodigestion of the gland with local inflammation [1]. It is a common cause of abdominal pain and admission to General Surgery wards. In the UK, the incidence is described as 56 per 100,000 people per annum with 50% cases due to gallstones, 25% due to alcohol and 25% because of other causes [2,3]. Some studies have postulated that COVID-19 may enter cells using ACE-2 (angiotensin-converting enzyme-2) receptors. As these receptors are abundantly expressed on pancreatic cells, this organ may be a target organ for the virus [4,5]. The COVID-PAN study has been the largest to date which described worse outcomes in patients with AP with coexistent COVID-19 in terms of 30-day mortality, severity of pancreatitis, length of stay and organ failure. In addition, this study also demonstrated a high proportion of patients with pancreatitis of unknown cause [6].

Our study focuses on patients presenting with AP during the initial six months of the pandemic to Altnagelvin Area Hospital in Derry/Londonderry, Northern Ireland. It aims to evaluate the baseline characteristics, incidence of concomitant COVID-19, outcomes, management and follow-up data in our catchment area population.

## Materials and methods

Study design

This was a retrospective cohort study of consecutive patients admitted with AP during the COVID-19 pandemic from 1st March, 2020 till 31st August, 2020 to Altnagelvin Area Hospital which is part of Western Health and Social Care Trust, Northern Ireland. Data were collected with a paper-based tool on which patients were anonymised with study-specific serial numbers and no patient identifiable data was required. This tool was similar to the data collection tool used by COVID-PAN study with some modifications to identify some locally applicable data. This study was registered with our local Audit and Quality Improvement department. A formal ethics approval or Institutional Review Board approval was not required as this was deemed a retrospective cohort study in line with recommendations from National Health Service Health Research Authority (NHS HRA) regulations.

Data collection

Patient case notes were reviewed for data collection. In addition, follow-up data were collected from review of Northern Ireland Electronic Care Record (NIECR) for all patients. Imaging was reviewed using the Northern Ireland Picture Archiving and Communications System (NIPACS) along with review of radiology reports. Data collection was completed between mid-December 2020 till the end of January, 2021 which provided ample time to obtain follow-up data as well.

Inclusion criteria

All patients with pancreatitis based on symptoms, amylase level three times above normal range and/or acute pancreatitis based on CT scan who were admitted irrespective of the aetiology were included in this study.

Exclusion criteria

Patients presenting with suggestive blood markers such as hyperamylasemia but no clinical and radiographic features of pancreatitis and other likely diagnosis were excluded from the study.

Definitions

SARS-CoV-2 infection was defined as a positive swab, positive CT thorax or a clinical diagnosis of symptomatic SARS-CoV-2 in patients for whom a swab test and CT scan were unavailable [6]. Pancreatitis of unknown aetiology was defined as patients who during this particular admission did not have a defined aetiology despite multiple investigations at the time of discharge/death [6]. Severity of pancreatitis was defined as per Revised Atlanta classification [7] whereby: a. Mild AP has no organ failure, local or systemic complications. b. Moderately severe AP is characterised by the presence of transient organ failure, local complications or exacerbation of co-morbid disease. c. Severe AP is defined as persistent organ failure, that is, organ failure >48 h. Eastern Cooperative Oncology Group (ECOG) score was used to describe baseline function of patients with values ranging from 0 to 4 (where 0 is fully independent and 4 is characterised by severe disability and poor function) [8]. Glasgow score was also obtained based on blood tests at the time of admission to risk stratify patients although this was done retrospectively as the scores were only calculated on admission for only two patients as per clinical notes despite the data being available [9]. Where an abdominal CT scan was available, the Modified CT Severity Index was used to classify the severity of AP radiologically as well [10]. CT scans were performed on patients with severe pancreatitis at admission or patients with AP. CT scans after one week of admission were also performed in cases of mild to moderate pancreatitis which either progressed to severe pancreatitis during inpatient stay or failed to improve significantly within one week of admission. Data variables collected: Several data variables such as demographics, blood tests, severity of AP (based on the revised Atlanta criteria), premorbid ECOG score, endoscopic or surgical interventions for drainage of pancreatic pseudocyst or walled-off necrosis and 30-day mortality were recorded. Follow-up data were also collected. Our questionnaire was based on the one used during COVID PAN study with minor amendments [6]. Demographic data included age, gender, smoking status and underlying co-morbidities such as previous acute pancreatitis, chronic obstructive pulmonary disease, chronic pancreatitis, hypertension, ischemic heart disease, diabetes mellitus, cerebrovascular accident and chronic kidney disease. Severity of acute pancreatitis was assessed using Glasgow score at admission (0-2 considered mild, score 3 or above considered severe pancreatitis), Revised Atlanta classification and modified CT severity index where applicable [11]. ITU admission data such as length of stay, level of support (including renal, respiratory or circulatory) required and patient outcomes at end of ITU stay were recorded. Renal support was defined as continuous veno-venous hemofiltration (CVVH) or hemodialysis. Respiratory support included high flow nasal oxygen, continuous positive airway pressure (CPAP), non-invasive ventilation (NIV) or intubation and ventilation. Circulatory support included the use or inotropes, vasopressors or both. Use of antibiotics and the rationale for their use were also recorded. Antibiotics were started according to local guidelines if patients had a source of infection such as respiratory, urinary, line-related, etc. Antibiotics were also started if there was concern that the patients were developing ascending cholangitis as well. Patients with acute pancreatitis only and no evidence of infection otherwise were not started on antibiotics. Data regarding follow-up and index or interval surgical procedures especially in the setting of gallstone pancreatitis was also noted.

Outcome measures

We collected outcome data on aetiology, ITU admission and length of stay, need for intervention (surgical, endoscopic or radiological), antibiotic use and local complications based on radiographic findings. Severity of acute pancreatitis was also noted as part of the study. Outcomes were compared to outcomes for COVID-19 negative group in COVID PAN [6] study due to extremely low incidence of AP with coexistent COVID-19 infection admitted to our centre. SPSS was used to compare our findings to findings of COVID-PAN study with odds ratio analysis.

## Results

Incidence of co-existent COVID-19

Forty-three patients were admitted with AP during our study period and the last date for inclusion into this study was set at 31st August, 2020. One patient (2.3%) had co-existent COVID-19 infection diagnosed by a swab test (PCR). Patient had only minor upper respiratory symptoms (cough, fever and mild dyspnea) and was admitted for concomitant ascending cholangitis with gallstone pancreatitis treated with intravenous antibiotics on this presentation. The patient had previously undergone a subtotal cholecystectomy in 2015. The patient subsequently underwent a completion cholecystectomy two months later after complete resolution of her COVID-19 infection. AP was classified as mild for this patient based on Revised Atlanta classification.

Patient demographics

Patient demographics data such as age, ethnicity and gender are detailed in Table 1.

**Table 1:** Description of patient demographics in acute pancreatitis patients.

Variable	Category	Patients	
		N=43	Summary
Age (mean +/- standard deviation in years)	-	43	63.13+/-16.35 range = 29-91
Sex	Male	43	25 (58.1%)
	Female		18 (41.9%)
Ethnicity	White	43	43 (100%)
	Asian		
	Black		
	Other		

Baseline characteristics: Hypertension was found to be the most common co-morbidity on review of the past medical history of patients. Many of our patients had multiple co-morbidities. Table 2 details the incidence of different co-morbidities in patients.

**Table 2:** Incidence of co-morbidities in acute pancreatitis patients. COPD: chronic obstructive pulmonary disease; HTN: hypertension; IHD: ischaemic heart disease; CKD: chronic kidney disease; CVA: cerebrovascular accident.

Incidence of co-morbidities	Conditions
Previous acute pancreatitis	12
COPD	6
HTN	22
IHD	8
Diabetes mellitus	4
CKD	4
Asthma	3
CVA	2

Baseline patient characteristics were noted including smoking status, COVID status at admission, BMI and ECOG scores which are detailed in Table 3. Smoking status was only available for 37 patients and BMI was only available for 36 patients.

**Table 3:** Baseline characteristics of pancreatitis patients. BMI: body mass index; ECOG score: Eastern Cooperative Oncology Group score.

Variable	Category	Patients	
		N=43	Summary
Smoker	No	37	30 (81.1 %)
	Yes		7 (18.9%)
COVID-19	Positive	43	1 (2.3%)
	Negative		42 (97.7%)
Premorbid ECOG	0	43	28 (65.1%)
	1		2 (4.6%)
	2		7 (16.3%)
	3 or 4		6 (13.9%)
BMI (at admission)		36	27.78 +/- 6.20

Severity of AP and aetiology

Severity of AP was Glasgow score was calculated at time of admission retrospectively based on patient laboratory results. Pancreatic necrosis was noted in three patients (7%). Eight patients (18.6%) had local complications of peripancreatic collections, out of whom 4 (50%) were reported to have acute pancreatic fluid collections and 4 (50%) patients were reported to have pseudocysts. One patient (2.3%) also had a splenic vein thrombus and the patient was anticoagulated thereafter. Twelve patients (27.9%) were noted to have hepatic steatosis based on imaging. These included three out of the four diabetic patients identified in the study.

Gallstones were noted to be the most common cause of pancreatitis in our study. Classification of severity of pancreatitis is further detailed in Table 4 based on Glasgow score on admission, Atlanta classification and MCTSI. Aetiology and other radiographic data are also detailed in this table.

**Table 4:** Aetiology, severity and radiographic features of acute pancreatitis patients. ARDS: acute respiratory distress syndrome.

Variable	Category	Patients	
		N=43	Summary
Aetiology	Gallstones	43	19 (44.1%)
	Alcohol		12 (27.9%)
	Post-ERCP		3 (6.9%)
	Unknown		8 (18.6 %)
	Other		1 (2.3%)
Atlanta classification	Mild	43	34 (79.1%)
	Moderate-severe		5 (11.6%)
	Severe		4 (9.3%)
ARDS	No	43	3 (7%)
	Yes		40 (93%)
Liver steatosis	No	43	31 (72.1%)
	Yes		12 (27.9%)
Glasgow score at admission	0-2	43	36 (83.7%)
	3 or more		7 (16.3%)
Index cholecystectomy	No	19	19 (100%)
	Yes		0 (0%)
Modified CT severity index	0-3 (mild)	39	32 (82%)
	4-6 (moderate)		7 (18%)
	7-10 (severe)		0

Outcomes

ITU admission was required by a total of five patients (11.62%). Among these, two patients required only cardiovascular support with inotropes whereas three patients had multiorgan failure and required cardiovascular support (with inotropes), respiratory support (two patients requiring high flow nasal cannula oxygen treatment and one requiring ventilation) and renal support (with haemodialysis). Two patients admitted to ITU had a fatal outcome and cause of death in both was multiorgan failure due to pancreatitis. Length of stay (LOS) in ITU was 1 to 51 days with a mean of 18.2 days. Median LOS was eight days in ITU.

Antibiotics were started in 21 patients (49%). Among these, only two patients had blood culture-proven bacteraemia (one had a GI source i.e., likely ascending cholangitis with concomitant acute pancreatitis and one had line infection). One patient was given antibiotics to cover for infective pancreatic necrosis, one was started on antibiotics as they were presumed to have splenic flexure colonic perforation at admission, one had pancreatic fluid collection cultures positive for E. faecium and one had possible infected pseudocyst on imaging. The remainder of the patients were started on antibiotics due to clinical suspicion of ascending cholangitis with acute pancreatitis.

Radiological, endoscopic or surgical interventions were required by a few patients as well. One patient required interventional radiology guided drainage of pancreatic fluid collections which grew E. faecium. Six patients underwent endoscopic retrograde cholangiopancreaticography (ERCP) during same admission. Out of these six patients, three had CBD stone extraction and three had stents inserted (one for suspicious CBD mass and two for CBD stones). The patient who had stent inserted for suspicious CBD mass developed post-ERCP pancreatitis. None of the patients with gallstone pancreatitis (total 19 patients) underwent index cholecystectomy during initial admission. One patient underwent laparotomy for possible splenic flexure perforation based on an initial CT scan report. He was found to have pancreatitis intra-operatively which was also confirmed on later CT scans in succeeding weeks after surgery (this patient also developed a gastropleural fistula in the ensuing months).

Length of stay (LOS) ranged from 1 to 113 days. Mean LOS and standard deviation were 7.49 +/- 6.74 days. Median LOS was 6 days. 30-day mortality was noted in three patients (7%). Among these, two died of multi-organ failure due to pancreatitis whereas one died of lung cancer (this patient was referred to oncology and palliative care teams for management once pancreatitis had clinically resolved). At time of discharge, 12 patients were diagnosed with alcohol-related pancreatitis, 19 with gallstone pancreatitis, 3 had pancreatitis post-ERCP, one had cystic fibrosis related pancreatitis, one had pancreatitis due to duodenal diverticulum and aetiology of pancreatitis was unknown in 7 patients. Exocrine pancreatic insufficiency was noted in 5 patients (11.62%) at end of hospital stay and they were given pancreatic enzyme replacement therapy.

Nine patients (21%) were readmitted with recurrent AP until January, 2021 (only one of these patients had gallstone pancreatitis). Furthermore, one patient was readmitted for cholecystitis, one for ascending cholangitis and one for biliary colic in the follow-up duration. Interval cholecystectomy was completed in only 4 out of 19 patients with gallstone pancreatitis (21%). In addition, three patients had ERCP after discharge (one for CBD stone extraction, one for CBD stent insertion and one for stent removal).

Comparison of outcomes

The outcomes of our study population were compared to outcomes of COVID-19 negative patients from COVID-PAN study. Our study had limitations in terms of a small number of patients who were eligible to be included. The number of COVID-19 positive patients was even lower. Therefore, none of our analyses reached statistical significance (p-value cutoff <0.05). Overall, we noted a higher incidence of ITU admission (11.63%), lower incidence of pancreatic necrosis (7% in our study), higher 30-day mortality (7%) and lower incidence of local complications (19.6%) for our patients. Secondary outcomes are detailed with comparison to COVID-PAN patients in Table 5.

**Table 5:** Comparison of outcomes of our study group with COVID-negative patients in COVID-PAN study.

Outcome	Category	Patients			
		N	n(%)	Incidence rate ratio	P-value
ITU admission	Altnagelvin	43	5 (11.63%)	1.52 (0.48-3.66)	0.355
	COVID-PAN (negative)	1616	123 (7.6%)	1	
Mortality in 30 days	Altnagelvin	43	3 (6.9%)	2.42 (0.48-7.54)	0.17
	COVID-PAN (negative)	1563	45 (2.9%)		
Length of stay (days)	Altnagelvin		7.49 +/- 6.74		
	COVID-PAN (negative)		4 (3,8)		
Necrosis	Altnagelvin	43	3 (7%)	0.53 (0.11-1.6)	0.28
	COVID-PAN (negative)	1367	177 (13.0%)		
Any local complication(e.g.,	Altnagelvin	43	8 (19.6%)	0.68 (0.29-1.35)	0.27
Collections)	COVID-PAN (negative)	1354	371 (27.4%)		
Portal or Splenic vein thrombus	Altnagelvin	43	1 (2.3%)	0.71 (0.02-4.31)	1.000
	COVID-PAN (negative)	1356	43 (3.2%)		
Organ failure	Altnagelvin	43	5 (11.62%)	1.80 (0.57-4.34)	0.22
	COVID-PAN (negative)	1597	103 (6.5%)		

## Discussion

This study was mostly focused to study the characteristics of AP in our target population in Derry/Londonderry area in Northern Ireland. There has only been one study on acute pancreatitis specific to Northern Ireland population in past by MK Heatly, et al in 1988 which focused on mortality data of the disease at that time [12]. Our study focuses in depth on the various patient characteristics and outcomes of the disease relevant to Derry/Londonderry area in Northern Ireland especially in setting of COVID-19 pandemic.

Acute pancreatitis is thought to be due to loss of intracellular and extracellular compartmentation, obstruction of pancreatic secretions and activation of pancreatic enzymes within the pancreas itself which leads to acinar cell damage and pancreatic inflammation [13]. Activated digestive enzymes can activate macrophages and lead to pro-inflammatory cytokine storms due to cytokines such as interleukin-1 (IL-1) and tumor necrosis factor-alpha (TNF-a) [14]. Marked inflammatory response can lead to systemic inflammatory response syndrome (SIRS). Excessive SIRS leads to multi-organ dysfunction syndrome (MODS) which carries high morbidity and mortality [15].

All patients included in our study were of White ethnicity given the population of Derry/Londonderry area is almost 98% White British or White Irish.

Patients with severe pancreatitis as per Atlanta classification were admitted to the ITU. Two of these five patients died and one had significant morbidity with repeated admissions and development of gastropleural fistula. This was in line with previous research which shows high mortality rate of more than 50% in patients admitted with pancreatitis to ITU setting [16]. Severity scoring for pancreatitis was also done utilizing the Modified CT severity index which builds on Balthazar score for stratifying severity of pancreatitis based on CT scan findings [17]. The MCTSI also incorporates pancreatic necrosis as part of evaluation of radiographic findings for AP [7]. Local complications associated with AP include acute pancreatic fluid collections (APFCs) and pancreatic pseudocysts once APFCs develop a well-enhancing wall after four weeks. Once these undergo necrosis, patients may develop acute necrotic collections (ANCs) or walled-off pancreatic necrosis (WOPNs) once ANCs mature and develop a wall after four weeks [18]. CT findings were evaluated to collect data on development of above complications in our study.

Aetiology of pancreatitis was found to be similar to the aetiology reported in most literature [3]. We did note that we were unable to ascertain the cause of AP in almost 16% of our patients despite outpatient investigations after discharge. Rare causes of acute pancreatitis namely duodenal diverticulum and cystic fibrosis were also observed in our study.

Prophylactic antibiotics are generally not recommended for mild AP. AP is usually self-limiting in its mild form. In severe AP, there are two phases. The early stage (first 14 days) comprises SIRS which may be complicated by multi-organ failure. In the late phase, usually 2-3 weeks after onset of AP patients may develop necrosis within in necrotic, inflamed pancreas [19]. In cases of severe AP with necrotizing pancreatitis, there is currently no consensus on antibiotic prophylaxis. If antibiotic prophylaxis is used, it is recommended to limit the duration to 7-14 days [20]. A Cochrane review in 2010 showed no benefit of antibiotics in preventing infection or reducing mortality, although use of imipenem significantly reduced pancreatic infection (studies included in this review were not adequately powered) [21]. In our study, patients noted to have necrotizing pancreatitis on CT scans were started on antibiotics. In addition, patients suspected to have concomitant ascending cholangitis were also given antibiotics.

Early studies such as one by J P Neoptoemos et al., in 1988 argued early ERCP should be done for all patients with severe gallstone pancreatitis within 72 hours of symptom onset [22]. However, a Cochrane review of seven studies in 2012 showed that there is no evidence to suggest that early routine ERCP in gallstone pancreatitis reduces mortality or the incidence of local or systemic complications regardless of disease severity. The Cochrane review did recommend early ERCP for patients with co-existing cholangitis or biliary obstruction [23]. A large multicenter randomized trial in Netherlands in 2020 also provided more evidence for the Cochrane review and recommended urgent ERCP for the above indications [24]. In our centre, we aimed early ERCP within 72 hours of symptom onset in acute gallstone pancreatitis patients with biliary obstruction or ascending cholangitis. However, in patients with hemodynamic instability and requiring ITU support or in the absence of above indications ERCP was deferred for later than 72 hours.

Recent studies such as by Noel et al., Wilson et al. and Giuffrida et al. recommended early cholecystectomy for patients presenting with mild gallstone pancreatitis within 48 hours of index admission with delayed cholecystectomy for severe cases once patients is clinically able to undergo the procedure [25-27]. During our study, theatre services globally were severely hampered due to the ongoing COVID-19 pandemic which led to none to the gallstone pancreatitis patients being offered index cholecystectomy. In our centre, it is recommended that we should aim to perform index cholecystectomy within 2 weeks in gallstone pancreatitis patients if pancreatitis resolves quickly. Otherwise, we should aim to perform interval cholecystectomy within 3 to 6 months as per local protocols. Only four patients underwent interval cholecystectomy within 6 months owing to delays and cancellation of all elective lists as well to manage the surge of COVID-19 pandemic. This trend of a decline in elective and emergency cholecystectomy has been recognized globally [26]. However, newer evidence and guidelines recommend that early cholecystectomy should be completed where possible when indications for this surgery are present. Campanile, et al. presented a multisociety position endorsing index cholecystectomy for acute cholecystitis and we shall aim in our centre that patients with mild gallstone pancreatitis are offered index cholecystectomy as well despite the restrictions due to the pandemic [28]. With easing restrictions and surging vaccination rates in Northern Ireland, we have seen an increase in index and elective cholecystectomies for all indications being performed in our department.

**Figure 1:**
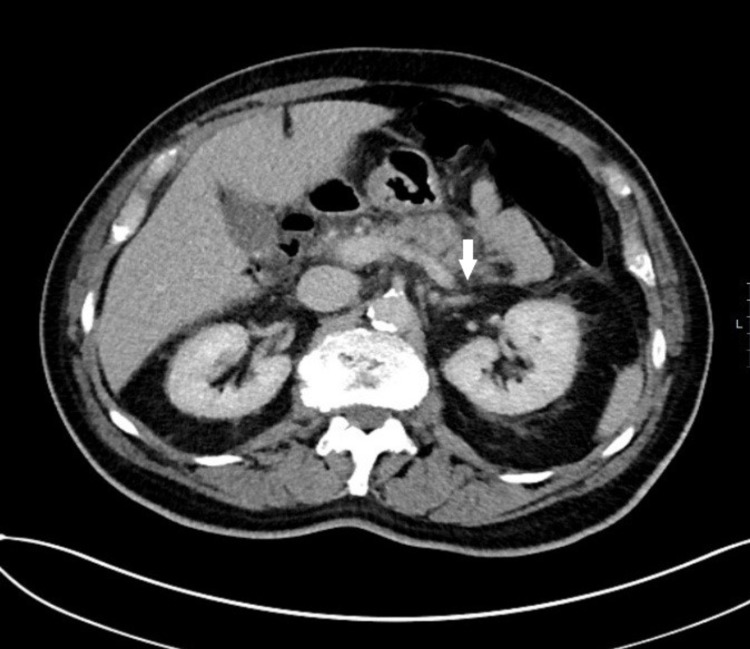
Axial CT scan showing acute pancreatitis. There is reduced enhancement in the region of the pancreatic tail and ill-defined pancreatic contours. Pancreatic parenchyma is not homogeneous in this region (white arrow).

**Figure 2:**
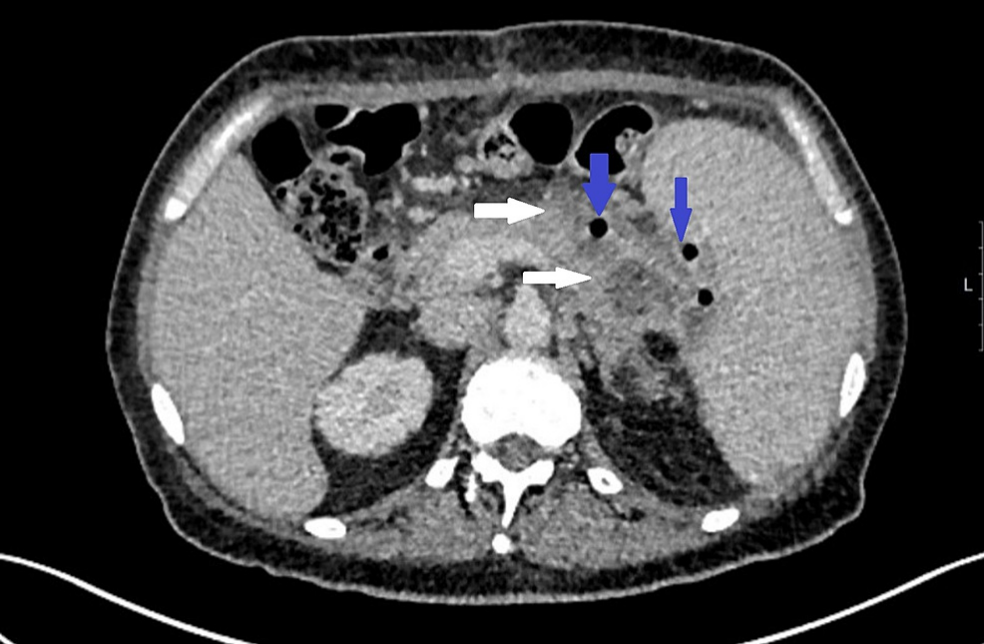
Axial CT scan showing acute necrotising pancreatitis. See non-enhancing low attenuation areas (white arrows) and extraluminal gas bubbles (blue arrows).

**Figure 3:**
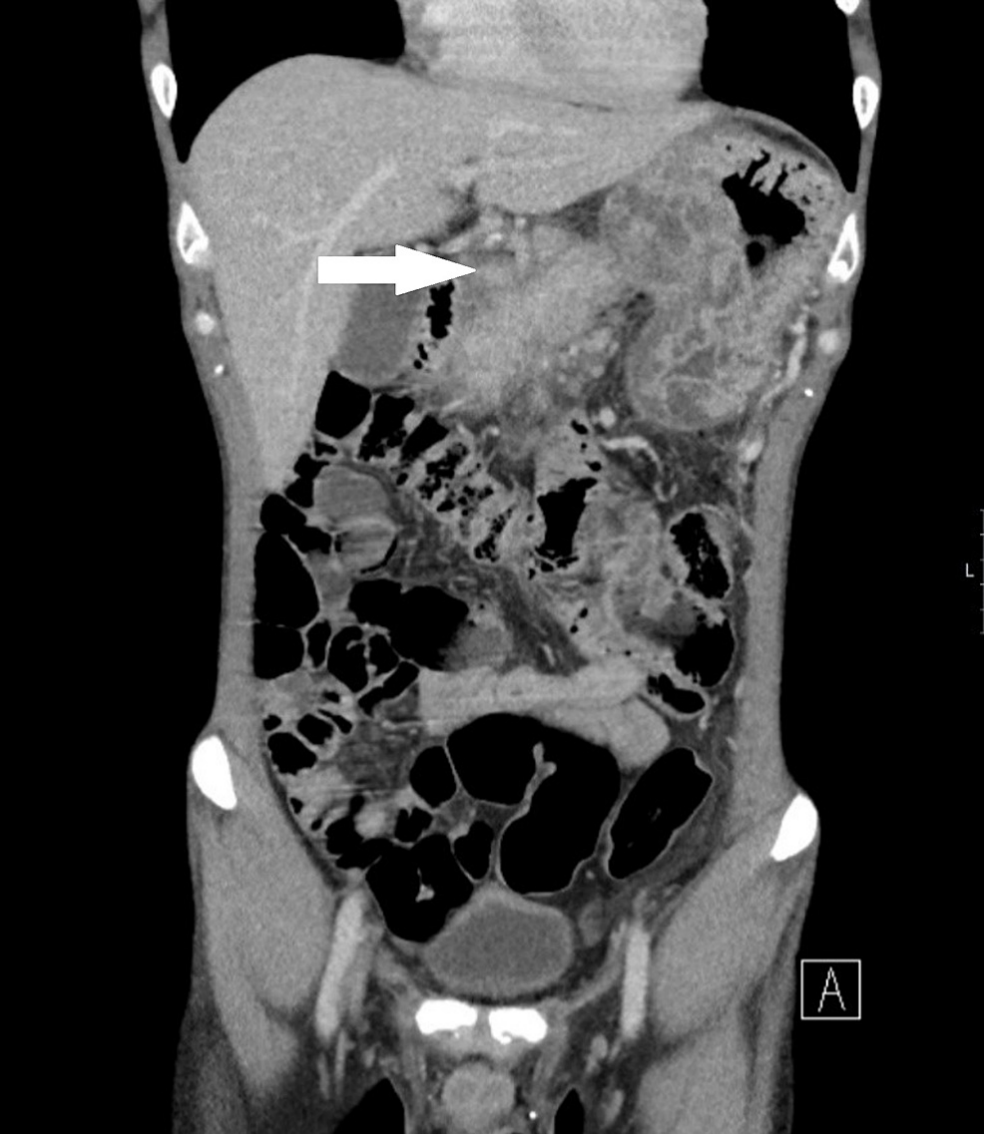
Coronal CT scan showing a large inflammatory peripancreatic fluid collections (white arrow).

**Figure 4:**
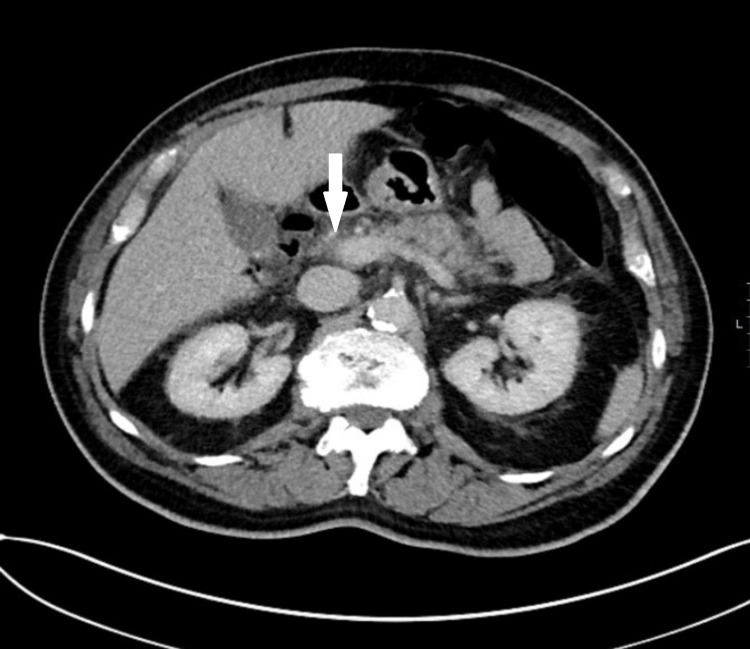
CT scan showing mild pancreatitis (see white arrow).

Some illustrations depicting pancreatitis and its complications in our study group are given below. Figure 1 shows a CT abdomen and pelvis of a pancreatitis patient with reduced enhancement in the region of the pancreatic tail. There is peripancreatic and mesenteric inflammatory stranding with multi-compartmental fluid. Figure 2 shows a CT abdomen and pelvis in a patient with features of necrotising pancreatitis. Figure 3 shows CT scan showing a large inflammatory phlegmon due to infected pancreatic pseudocyst with possible infiltration into the stomach. Figure 4 shows mild acute pancreatitis on a CT scan.

## Conclusions

In conclusion, we noted that although hardly any patients with acute pancreatitis had co-existent COVID-19 infection in our hospital, the pandemic had a severely adverse effect on service delivery. There was significant number of patients presenting with pancreatitis of unknown aetiology in this duration. Most of the patients had mild AP based on Revised Atlanta classification. Our study has several limitations as the number of patients who presented with acute pancreatitis during first wave of COVID-19 was very small. In addition, only one patient with acute pancreatitis was noted to be positive for COVID-19. We aim to conduct further reviews in our hospital during the second COVID-19 wave as well. Further larger-scale multicentre studies will also be needed to ascertain longer-term effects of the pandemic on acute pancreatitis patients and their management.
